# Influence of rebubbling on anterior segment parameters and refractive outcomes in eyes with DMEK for Fuchs endothelial dystrophy

**DOI:** 10.1007/s00417-021-05114-2

**Published:** 2021-04-06

**Authors:** Bishr Agha, Raimund Forster, Thomas Kohnen, Ingo Schmack

**Affiliations:** Department of Ophthalmology, Goethe-University, Theodor-Stern-Kai 7, 60590 Frankfurt am Main, Germany

**Keywords:** Rebubbling, Graft detachment, Descemet membrane endothelial keratoplasty (DMEK), Fuchs endothelial dystrophy (FED), Endothelial cell density (ECD)

## Abstract

**Purpose:**

To evaluate the potential impact of rebubbling on the anterior segment parameters and refractive outcomes in patients with graft detachment following uneventful DMEK for Fuchs endothelial dystrophy (FED).

**Methods:**

Retrospective institutional cohort study of comparing 34 eyes of 31 patients with rebubbling for graft detachment following Descemet membrane endothelial keratoplasty (DMEK) to 33 eyes of 28 patients with uneventful DMEK. Main outcome parameters were various corneal parameters obtained by Scheimpflug imaging, refractive outcome, corrected distance visual acuity (CDVA), and endothelial cell density (ECD).

**Results:**

Anterior and posterior corneal astigmatism, corneal densitometry, central corneal thickness, and anterior chamber depth and volume showed no significant differences. Preoperative distribution of astigmatism axis orientations showed a high proportion of anterior corneal with-the-rule astigmatism (71%) in eyes requiring rebubbling. Mean postoperative cylinder in the rebubbling group (1.21 ± 0.85 D) was significantly higher compared to the controls (*p* = 0.04), while differences in spherical equivalent (SE) were insignificant (*p* = 0.24). Postoperative CDVA was 0.11 ± 0.11 in the control group compared to 0.21 ± 0.17 in the rebubbling group (*p* = 0.03). Eyes with subsequent rebubbling demonstrated a significantly higher endothelial cell loss (56% versus 37%) (*p* < 0.001).

**Conclusion:**

Apart from higher cylinder values, refractive outcome and corneal parameters assessed by Scheimpflug imaging were comparable in eyes with rebubbling and controls. However, a reduced visual acuity and an increased endothelial cell loss should be taken into consideration prior to rebubbling especially in eyes with circumscribed graft detachment.

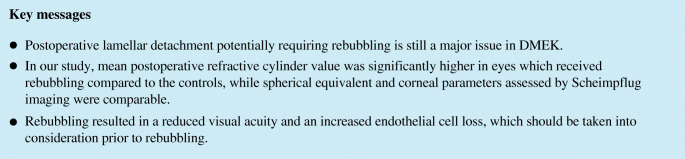



## Introduction

Patients suffering from visual loss secondary to endothelial cell dysfunction can effectively be treated by posterior lamellar keratoplasty (i.e., Descemet stripping automated endothelial keratoplasty (DSAEK) or Descemet membrane endothelial keratoplasty (DMEK)) [1, 2]. Compared to penetrating keratoplasty, posterior lamellar endothelial transplantation is characterized by several advantages like faster visual recovery, better functional outcome, less postoperative corneal astigmatism, and lower risk of graft rejection [3–5]. In contrast to DSAEK, DMEK displays less posterior corneal higher-order aberrations related with a higher patient satisfaction rate [6, 7]. Nevertheless, postoperative lamellar detachment is still a major issue in DMEK often requiring intracameral air and/or gas injection (rebubbling) [8]. Among the available tamponades, 20% sulfur hexafluoride (SF6) seems to be most effective without obvious side effects on the corneal endothelium [9]. However, there are still concerns about a potentially induced endothelial cell loss and impaired visual outcome secondary to rebubbling [10, 11].

Currently, no general agreement exists in regard to the exact indication and perfect timing of rebubbling. There were even single cases of spontaneous corneal clearance despite graft detachment after DMEK reported [12]. While there are studies that have attributed the need for rebubbling in regard to several risk factors [13–15], there are currently limited data available on corneal topographic changes after rebubbling and its impact on the final postoperative refraction.

The purpose of our study was to address the abovementioned parameters in eyes undergoing rebubbling for graft detachment after DMEK. In addition, further information should be provided for the decision-making process whether or not to perform a rebubbling procedure in patients with detached DMEK lamellae.

## Patients and methods

### Study design and data collection

Medical records of patients undergoing rebubbling for graft detachment after uneventful DMEK surgery for FED between November 2016 and April 2018 were reviewed. Patients with a sufficient follow-up time of at least 3 months after rebubbling were included (rebubbling group). Eyes with uneventful DMEK and primarily completely attached grafts served as controls (control group). The age-matched control group was composed of eyes with a comparable preoperative corrected distance visual acuity (CDVA) and follow-up time. Both groups were composed of phakic and pseudophakic patients. However, all phakic patients underwent simultaneous uneventful cataract and DMEK surgery (triple DMEK) due to significant lens opacifications.

Overall, 34 eyes of 31 patients requiring rebubbling for graft detachment after DMEK met the abovementioned inclusion criteria. The control group was composed of 33 eyes of 28 patients. Eyes with major extracorneal visual limitations (age-related macular degeneration, advanced glaucoma, and amblyopia) were excluded from further evaluation in regard to CDVA (rebubbling group: *n* = 7 versus control group: *n* = 5). The mean follow-up time was 9.2 ± 4.1 months (range: 3–18 months) for the rebubbling group and 10.9 ± 4.0 months (range: 4–18 months) for the control group.

In situations where both eyes of a patient were affected, the left and right eyes were assumed to be independent.

The study protocol was approved by the institutional review board of the Goethe-University and adhered to the tenets of the declaration of Helsinki.

### Surgical technique

#### DMEK and postoperative treatment

DMEK surgeries and subsequent rebubbling procedures were routinely carried out by two experienced surgeons (TK, IS) at the Department of Ophthalmology, Goethe-University, Frankfurt, Germany. Preoperatively, donor corneas were prepared as previously described by Melles et al. [16]. The graft diameter ranged from 7.75 to 8.0 mm (based on the white-to-white distance of the eye). Grafts were stained with trypan blue, stored in a glass cartridge (Geuder AG, Heidelberg, Germany). The diseased Descemet membrane was subsequently removed within a diameter of 9.0 mm. Once the DMEK graft was injected via a 2.2-mm corneal incision, unfolded, and centered, 20% sulfur hexafluoride (SF6) gas was installed between the iris and DMEK graft to achieve complete graft adherence. Anterior chamber gas fill ranged between 80 and 90%. A peripheral iridectomy at 6 o’clock was performed in each patient during DMEK surgery to prevent a postoperative pupillary block due to misplacement of 20% sulfur hexafluoride (SF6) gas behind the iris. For the first 2–3 postoperative days, patients were asked to predominantly stay in a supine position.

Postoperatively, patients received a standardized treatment protocol composed of topical antibiotics (ofloxacin eyes drops, applied 4 times a day for 1 week), miotics (pilocarpine 1% eye drops, applied 3 times a day as long as the anterior chamber was filled with gas), and steroids (dexamethasone eye drops, applied 6 times a day for the first 8 weeks). Three months after surgery, topical steroids were slowly tapered down to a dose of one application per day.

#### Indication of rebubbling and determination of success

**Fig. 1:**
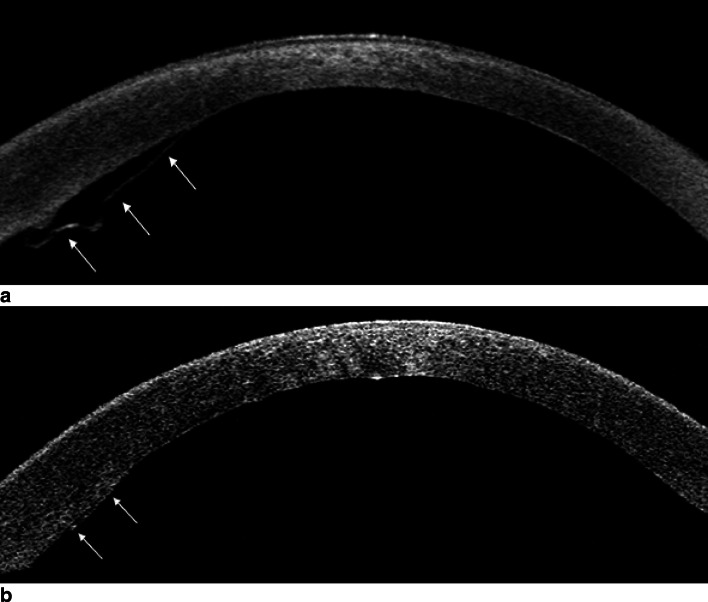
Representative anterior segment optical coherence tomography images showing DMEK grafts with partial detachment (white arrows). **a** Detached DMEK graft with adjacent corneal stromal thickening and a subtle peripheral inward fold meeting criteria for rebubbling (more than three clock hours). **b** Circumscribed area of a slightly detached DMEK graft suitable for observation for spontaneous attachment

Indications for rebubbling were based on slit-lamp examination and anterior segment optical coherence tomography (Visante OCT, Carl Zeiss, Meditec, Jena, Germany). All patients with a partial graft detachment of more than one third of the graft diameter or more than three clock hours and clinical signs of stromal or epithelial edema were scheduled for rebubbling. Figure 1 shows representative anterior segment OCT images of different graft detachments. Rebubbling procedures were performed under topical anesthesia. If possible, the original side ports of the previous DMEK surgery were reopened with a blunt speculum. Subsequently, a 30-gauge cannula was inserted in the anterior chamber, and 20% SF6 gas was injected between the iris and the detached DMEK lamella until the anterior chamber showed a gas fill of about 80 and 90%. Intraocular pressure control was performed between 1 and 2 h after surgery. The postoperative therapy was identical to those of the previous DMEK surgery. Rebubbling procedures were defined as successful when complete graft attachment and resolution of corneal edema was achieved once the entire gas was diminished.

#### Preoperative and postoperative assessment

Demographic data, visual acuity, refraction, and ocular comorbidities were obtained from the medical records. In addition, we evaluated the number of eyes requiring more than one rebubbling and the number of patients requiring secondary keratoplasty (repeat DMEK or penetrating keratoplasty) after rebubbling. However, eyes scheduled for secondary keratoplasty were excluded from final evaluation. Postoperative analysis included endothelial cell density (ECD) (Oculus/Nidek CEM-530, Oculus GmbH, Wetzlar, Germany), central corneal thickness (CCT), corneal front and back astigmatism, average keratometry readings of the anterior (KmF) and posterior surface (KmB), anterior chamber depth (ACD) and volume (ACV), corneal volume (CV), and corneal densitometry (CD) obtained by Scheimpflug tomography (Pentacam AXL, Oculus, Wetzlar, Germany).

Definitions used for the analysis of axis distribution of corneal astigmatism were for anterior astigmatism: with-the-rule (WTR: steep meridian within 60–120°), against-the-rule (ATR: steep meridian within 0–30° or 150–180°), and oblique (steep meridian within 30–60° or 120–150°). For posterior astigmatism: WTR (steep meridian within 0–30° or 150–180°), ATR (steep meridian within 60–120°), the remaining astigmatism was classified as oblique astigmatism [17].

Corneal light backscatter, a common parameter describing corneal clarity, was expressed in grayscale units (GSU) ranging from 0 (100% transparent) to 100 (completely opaque). It was calculated for 4 concentric corneal annular zones (0–2-mm zone, 2–6-mm zone, 6–10-mm zone, and 10–12-mm zone) and 3 corneal layers (anterior, central and posterior layer) as well as the total corneal layer (from epithelium to endothelium). In our study, we analyzed the values of the total layer for the 0–2, 2–6, and 6–10-mm zones. The 10–12-mm zone was not further evaluated because it was beyond the graft diameter, and its reproducibility was assumed to be weak as already shown by previous studies [18].

### Statistical analysis

Statistical analysis was performed using Excel for Mac (version 15.37, Microsoft, Inc., Redmond, WA, USA) and SPSS version 25 (IBM Corp., Armonk, NY, USA). Intergroup comparative statistics were determined by using the independent-sample *t*-test or Mann-Whitney *U* test. Differences of parameters within the groups were assessed by *t*-test for paired values or a two-sided Wilcoxon signed-rank test, depending on the normality of data distribution. A *p* value less than 0.05 was considered statistically significant.

## Results

The mean age (± SD) at the time of DMEK surgery was 70.1 ± 7.8 years (range: 51–82 years) in the rebubbling group and 70.7 ± 7.4 years (range: 53–83 years) in the control group. Patients and donor characteristics are summarized in Table 1.

**Table 1:** Patient and donor tissue characteristics for the rebubbling and control group. *ECD* endothelial cell density. Mean ± SD (range)

	Rebubbling group	Control group
Eyes/patients	34/31	33/28
Sex (m/f)	13/18	12/16
Recipient age (years)	70.1 ± 7.8 (51–82)	70.7 ± 7.4 (53–83)
Triple DMEK (*n*)	27	17
Follow-up time (months)	9.2 ± 4.1 (3–18)	10.9 ± 4.0 (4–18)
Donor data		
Donor age (years)	74.8 ± 8.2 (55–88)	77.1 ± 8.7 (56–87)
Storage time (days)	19.6 ± 4.1 (11–26)	16.4 ± 7.5 (7–28)
ECD graft (cells/mm^2^)	2756 ± 333 (2306–3600)	2886 ± 336 (2300–3500)

The median time frame between initial DMEK surgery and rebubbling was 7.5 days ranging from 1 to 67 days. More than a single rebubbling procedure was performed in 11 eyes (2–times: *n* = 10, 3-times: *n* = 1).

Despite rebubbling, secondary keratoplasty became necessary in 4 eyes of 4 patients (11.8%). In 3 patients, repeat DMEK was performed 22, 92, and 122 days after initial DMEK. One patient required penetrating keratoplasty due to graft failure and corneal scarring 50 days after DMEK and rebubbling. Two of the patients requiring secondary keratoplasty, including the patient that received a penetrating keratoplasty, had previously more than one rebubbling procedure.

Preoperatively, CDVA did not differ significantly between the two groups (0.48 ± 0.3 rebubbling group; 0.39 ± 0.21 control group; *p* = 0.17). Patients of both groups showed an improvement in visual acuity after DMEK. However, the final postoperative CDVA was significantly better in the control group (0.11 ± 0.11 compared to the rebubbling group (0.21 ± 0.17) (*p* = 0.03).

Spherical equivalent (SE) changed from −0.31 ± 1.77 to −0.59 ± 1.06 D in the rebubbling group and from −0.24 ± 2.53 to −0.19 ± 1.21 D in the control group. There was no significant difference in SE between both groups pre- and postoperatively (*p* = 0.81 and 0.24, respectively). In addition, cylindrical values did not differ significantly between the groups, preoperatively (*p* = 0.19). Postoperatively, both groups demonstrated a reduction of cylinder (rebubbling group, 1.21 ± 0.85 D; control group, 0.78 ± 0.53 D), which resulted in a significant intergroup difference (*p* = 0.04) (see also Table 2).

**Table 2:** Preoperative and postoperative visual acuity and refraction, mean ± SD (range). *CDVA* corrected distance visual acuity, *logMAR* logarithm of the minimum angle of resolution, *Cyl* cylinder, *SE* spherical equivalent, *D* diopters. Mean ± SD (range)

	Rebubbling group	Control group	*p*
Preoperative			
CDVA (logMAR)	0.48 ± 0.3 (0.05–1.3)	0.39 ± 0.21 (0.1–1)	0.17
SE (D)	−0.31 ± 1.77 (−4.5–2.75)	−0.24 ± 2.53 (−5.88–4.75)	0.81
Cyl (*D*)	1.52 ± 1.41 (0–7.5)	1.0 ± 0.58 (0–2.75)	0.19
Postoperative			
CDVA (logMAR)	0.21 ± 0.17 (−0.1–0.6)	0.11 ± 0.11 (−0.1–0.4)	0.03
SE (D)	−0.59 ± 1.06 (−3.1–0.75)	−0.19 ± 1.21 (−3.75–1.75)	0.24
Cyl (D)	1.21 ± 0.85 (0–3.5)	0.78 ± 0.53 (0–2.5)	0.04

In both groups, anterior corneal astigmatism remained fairly stable after DMEK. Values were slightly higher in eyes with rebubbling (1.44 ± 1.32 D preoperatively and 1.53 ± 1.26 D postoperatively) compared to controls (1.20 ± 1.16 D preoperatively and 1.12 ± 0.61 D postoperatively). Nevertheless, differences between both groups were not statistic significant at either time point (*p* = 0.26 and 0.19). In addition, comparison of posterior corneal astigmatism did not reveal any statistic significant differences between the two groups before and after DMEK (Table 3).

**Table 3:** Scheimpflug parameters before (preoperative) and after (postoperative) DMEK. *CCT* central corneal thickness, *CAant* anterior corneal astigmatism, *CApost* posterior corneal astigmatism, *KmF* average keratometry readings of the anterior surface, *KmB* keratometry readings of the posterior surface, *CV* corneal volume, A*CV* anterior chamber volume, *ACD* anterior chamber depth, *CD* corneal densitometry, *D* diopters

	Rebubbling group Mean ± SD (range)	Control group Mean ± SD (range)	*p*
Preoperative			
CCT (μm)	617.5 ± 40.4 (540–730)	639.2 ± 70.9 (532–866)	0.25
CAant (D)	1.44 ± 1.32 (0.2–6.4)	1.20 ± 1.26 (0–7)	0.26
CApost (D)	0.37 ± 0.25 (0.1–1.2)	0.54 ± 0.42 (0.1–2.1)	0.12
KmF (D)	44.0 ± 1.6 (39.7–48)	43.6 ± 3.2 (36.4–56.8)	0.54
KmB (D)	−5.9 ± 0.33 (−6.6–−5.2)	−5.7 ± 0.6 (−7.8–−3.9)	0.09
CV (mm^3^)	62.2 ± 2.9 (55.2–67.2)	63.5 ± 6.3 (53.1–92)	0.44
ACV (mm^3^)	144.6 ± 47.2 (53–236)	161.1 ± 38.8 (102–241)	0.12
ACD (mm)	2.78 ± 0.72 (1.35–4.65)	3.07 ± 0.94 (1.41–5.7)	0.23
CD (zone)			
0–2 mm	22.3 ± 5.4 (14.2–36.3)	23.5 ± 6.4 (13.8–38.6)	0.45
2–6 mm	19.9 ± 4.1 (14.7–34.6)	20.6 ± 5.5 (12.5–36.3)	0.75
6–10 mm	30.2 ± 7.0 (19.4–45.9)	27.3 ± 7.4 (14.1–45)	0.09
Postoperative			
CCT (μm)	545.9 ± 40.8 (434–631)	539.6 ± 42.4 (461–635)	0.46
CAant (D)	1.53 ± 1.26 (0.1–6.7)	1.12 ± 0.61 (0.2–2.7)	0.19
CApost (D)	0.42 ± 0.3 (0–1.5)	0.4 ± 0.32 (0.1–0.9)	0.58
KmF (D)	43.8 ± 1.3 (41–45.8)	43.0 ± 1.8 (38.2–46.6)	0.06
KmB (D)	−6.4 ± 0.24 (−6.9–−6)	−6.3 ± 0.33 (−7–−5.4)	0.06
CV (mm^3^)	61.7 ± 4.0 (53.4–73.7)	61.0 ± 4.9 (52.3–74.1)	0.42
ACV (mm^3^)	198.8 ± 23.2 (128–241)	192.7 ± 26.2 (154–263)	0.11
ACD (mm)	4.37 ± 0.51 (2.64–5.21)	4.35 ± 0.7 (3.02–6.28)	0.67
CD (zone)			
0–2 mm	17.2 ± 3.0 (12.5–24.1)	17.3 ± 3.2 (12.2–24.8)	0.96
2–6 mm	17.6 ± 3.4 (11.5–23.8)	17.5 ± 3.9 (11.9–30.9)	0.64
6–10 mm	29.5 ± 7.0 (18.9–45.3)	26.8 ± 6.3 (16.7–40.9)	0.15

**Fig. 2:**
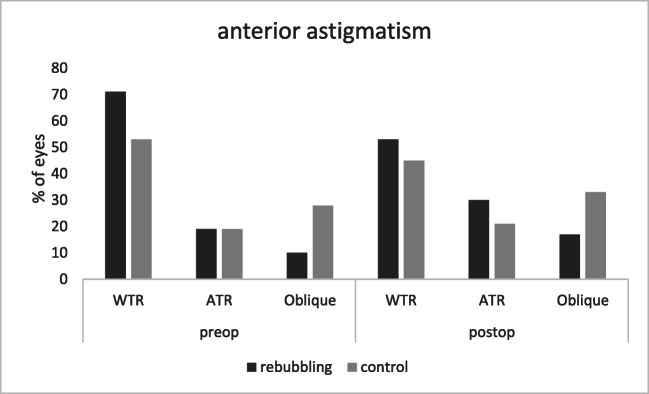
Axis orientations (% of eyes) of anterior corneal astigmatism before DMEK (preop) and after follow-up time (postop) in the rebubbling and control group. *WTR* with-the-rule astigmatism, *ATR* against-the-rule astigmatism, *Oblique* oblique astigmatism

**Fig. 3:**
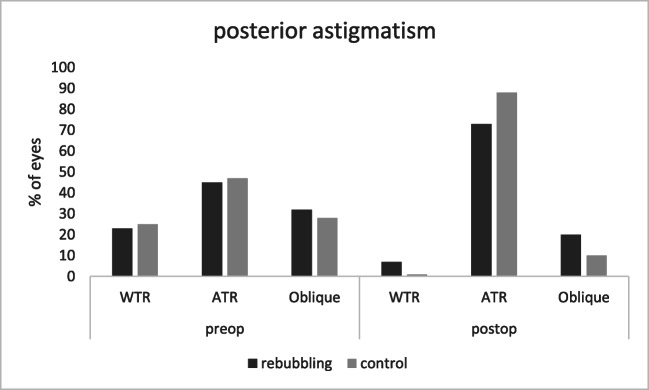
Axis orientations (% of eyes) of posterior corneal astigmatism before DMEK (preop) and after follow-up time (postop) in the rebubbling and control group. *ATR* against-the-rule astigmatism, *Oblique* oblique astigmatism, *WTR*, with-the-rule astigmatism

Regarding the distribution of axis orientations of anterior corneal astigmatism, there was a higher preoperative proportion of WTR astigmatism in eyes that required rebubbling after DMEK surgery (71%) than in the control group (53%). Preoperatively, oblique astigmatism was present in 10% of eyes of the rebubbling group and in 28% of eyes of the control group. The proportion of eyes with ATR astigmatism was similar in both groups (Fig. 2). Postoperatively, there was mainly a shift towards ATR and oblique astigmatism in the rebubbling group. In contrast, the distribution of axis orientations remained almost constant in the control group. Axis distributions of posterior astigmatism were similar between both groups and showed a shift towards predominantly ATR astigmatism, postoperatively (Fig. 3).

Central corneal thickness decreased from 617.5 ± 40.4 before DMEK to 545.9 ± 40.8 μm in the rebubbling group and from 639.2 ± 70.9 to 539.6 ± 42.4 μm in the control group. Intergroup comparison showed no significant difference in regard to CCT measurements (*p* = 0.25).

There was a tendency towards lower preoperative ACV and ACD values in the rebubbling group (144.6 ± 47.2 mm^3^ and 2.78 ± 0.72 mm) compared to the control group (161.1 ± 38.8 mm^3^ and 3.07 ± 0.94 mm), which was statistically not significant (*p* = 0.12 for ACV and *p* = 0.23 for ACD). Postoperatively, both groups demonstrated an increase in ACV and ACD, which was more pronounced in eyes that underwent rebubbling (Table 3).

Corneal densitometry decreased in all 3 corneal annular zones after surgery. Regarding intergroup comparison, there were no significant differences observed.

**Fig. 4:**
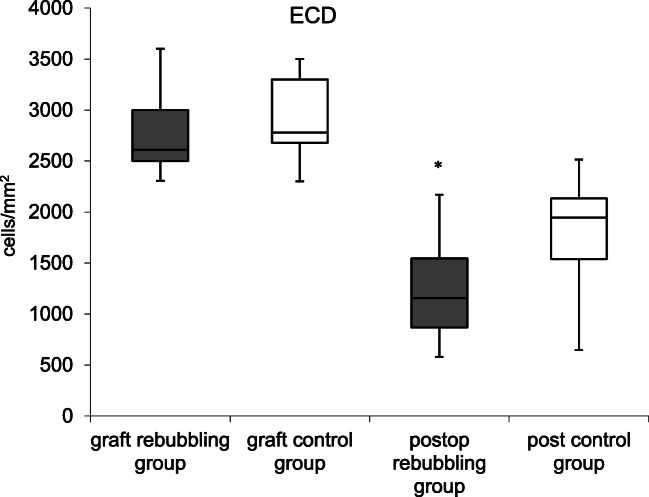
ECD of graft and after DMEK in the rebubbling and control group (box plot showing median, interquartile range, and full range of data). *p < 0.05

**Fig. 5:**
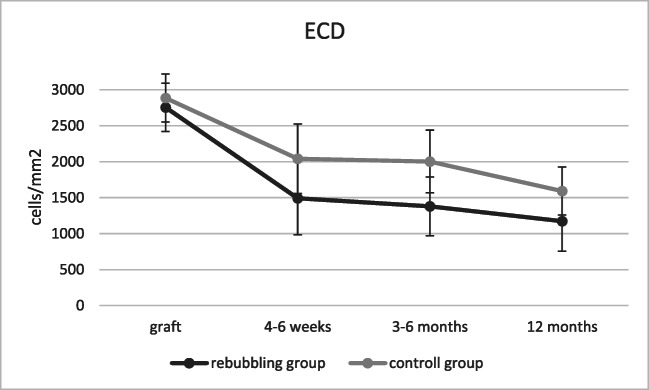
Changes in endothelial cell density (ECD, cells/mm^2^). Error bars depicting ± SD

The ECD of donor grafts was comparable in both groups (*p* = 0.08). During follow-up, there was a decrease of ECD from 2756 ± 333 to 1216 ± 423 cells/mm^2^ in the rebubbling group and from 2886 ± 336 to 1815 ± 498 cells/mm^2^ in the control group (Fig. 4). The resulting endothekial cell loss was 56% in the rebubbling group and 37% in the control group, respectively. The mean ECD was statistically significant lower in eyes with rebubbling compared to controls (*p* < 0.001), even when only the eyes with a single rebubbling were analyzed. The ECD showed a marked decrease 4–6 weeks after surgery and the rebubbling group demonstrated lower ECD during a 12 months follow-up period (Fig. 5).

## Discussion

In the present study, we evaluated the potential impact of rebubbling on the refractive and visual outcome in eyes with FED. In addition, corneal parameters assessed by Scheimpflug tomography like corneal astigmatism and its axis distribution as well as changes in ECD were analyzed.

We found that the corneal topographic parameters did not differ significantly after rebubbling compared to uncomplicated DMEK cases. In terms of spherical equivalent, postoperative refractive outcome was comparable in the eyes which received rebubbling and controls. However, cylindrical error remained > 1 D in the rebubbling group and was significantly higher compared to the control group at the follow-up. Although there was a tendency towards higher anterior corneal astigmatism values in the rebubbling group before and after DMEK, differences were statistically not significant.

It is already known that the distribution of astigmatism in FED patients differs from the normal population. A study by Miyake et al. reported that 50% of healthy subjects between 60 and 69 years demonstrate anterior WTR astigmatism; in patients between 70 and 79 years, WTR astigmatism was found in 43.3% [19]. In contrast, Yokogawa et al. reports WTR in 61.7% of patients with FED [20]. In our study cohort, 71% of eyes that eventually required rebubbling demonstrated preoperative WTR astigmatism compared to only 53% in the control group. This suggests that eyes which require rebubbling might demonstrate a higher preoperative proportion of WTR astigmatism. After rebubbling, axis orientation of astigmatism was more equal in both groups due to a decrease in the proportion of WTR astigmatism in the rebubbling group. This could either be surgically induced, dependent on the side-port used for the rebubbling procedure, or be an effect of corneal edema resolution and CCT reduction after complete detachment of the DMEK lamella.

Corneal densitometry is a well-established parameter to quantify corneal transparency. It has been reported that postoperative visual acuity correlates well with CD [21, 22]. Our analysis showed a comparable postoperative decrease in CD in all annular zones (0–2, 2–6, and 6–10 mm) in both groups. Since preoperative values were similar in the rebubbling and control group, the observed differences in the outcome in regard to visual acuity cannot be attributed to higher preoperative CD or subsequent morphologic changes in the rebubbling group. Our findings are congruous with a previous study which evaluated the effect of rebubbling on CD [23]. The authors did not find an increase of CD in the central cornea induced by rebubbling compared to a control group [23]. Furthermore, we did not observe structural abnormalities like microfolds of the DMEK lamella or significant stromal edema in the periphery of eyes which underwent rebubbling.

Gerber-Hollbach and co-workers concluded that rebubbling results in similar visual outcomes as in uncomplicated DMEK [11]. In contrast, we found a significantly lower postoperative CDVA in eyes with rebubbling compared to DMEK eyes without rebubbling (*p* = 0.03). Apart from slightly different indications for rebubbling, time interval between previous DMEK and rebubbling, and the follow-up after rebubbling were different from our study. In detail, rebubbling procedures were performed on average 25 days after DMEK in the study by Gerber-Hollbach, which is later than in our study (7.5 days). Additionally, postoperative follow-up was limited to 6 months compared to a mean follow-up of 9.2 (± 4.1) months in our study. It remains to be further investigated which factors contribute to a lower CDVA in patients that received rebubbling as it was found in our study.

In our study cohort, postoperative evaluation of ECD demonstrated a significant decrease in the rebubbling compared to the control group (*p* < 0.001). We found this difference also when only eyes which received a single rebubbling were analyzed. Postoperative ECL is a critical parameter while evaluating a patient for rebubbling due to a detached DMEK lamella since intraocular manipulation and or injection of SF6 gas is of potential harm to the sensitive corneal endothelium. The aforementioned study by Gerber-Hollbach detected a significant ECL (54% after 6 months follow-up) [11], which is congruous with findings reported by Lazaridis et al. [23]. Feng and colleagues also demonstrated that repeat intracameral air injections resulted in a significant decrease in ECD [10].

Apart from the criteria for indicating additional gas injections, optimal timing for the procedure has still to be defined. It is generally accepted that early rebubbling is favorable in regard to the clinical outcome, graft stiffness, and stromal fibrosis due to long-standing corneal edema complicating the procedure [8, 11]. Interestingly, we found lower preoperative ACD and ACV values in the eyes of the rebubbling group that could not be attributed to lens status or hyperopia. Although differences were not significant compared to the control group, reduced ACV might be at least a risk factor for additional endothelial cell damage during graft positioning or insufficient gas-bubble support, which has been reported to be associated with graft detachment [24].

In conclusion, corneal topographic and anterior segment parameters as well as the refractive outcome are comparable in eyes requiring rebubbling and those with uncomplicated DMEK. However, rebubbling shows to negatively influence the final visual outcome and the overall endothelial cell function potentially resulting in a poorer long-term prognosis of transplant. We are aware that our findings are limited by the small sample size and retrospective character of our study. Prospective studies might be helpful to further evaluate indication criteria and outcomes following rebubbling for graft detachment after DMEK. Nevertheless, rebubbling should be considered carefully, especially in DMEK eyes with only circumscribed graft detachment.
